# Establishment and characterization of canine mammary tumors 622: A novel luminal B CMT cell line exhibiting partial epithelial–mesenchymal transition and intermediate drug sensitivity

**DOI:** 10.14202/vetworld.2025.3306-3321

**Published:** 2025-11-06

**Authors:** Qingyang Peng, Shengjun Ma, Zihao Lu, Jiaqi Shi, Qingyu Zheng, Tao Zhang

**Affiliations:** Department of Traditional Chinese Veterinary Medicine, College of Veterinary Medicine, Anhui Agricultural University, Hefei, 230031, China

**Keywords:** canine mammary tumor, cell line establishment, epithelial–mesenchymal transition, luminal B subtype, osthole sensitivity, xenograft model

## Abstract

**Background and Aim:**

Canine mammary tumors (CMTs) serve as valuable comparative models for human breast cancer (HBC) owing to their shared biological and molecular features. However, well-defined cell lines representing the luminal B subtype remain limited. This study aimed to establish and characterize a novel CMT cell line, designated CMT-622, to expand available *in vitro* models for luminal B breast cancer research.

**Materials and Methods:**

Primary tumor tissue was collected from an 11-year-old female dog diagnosed with high-grade mammary carcinoma (T3N1M0). Tumor cells were isolated using enzymatic digestion and differential adhesion. Morphological, cytogenetic, and immunophenotypic characteristics were assessed using hematoxylin-eosin staining, immunohistochemistry, and immunofluorescence. Growth kinetics, clonogenicity, and chromosomal analyses were performed, and tumorigenicity was evaluated through xenograft assays in nude mice. Drug sensitivity and apoptosis were compared with two existing CMT lines (CMT-1211 and CMT-n7) using the cell counting kit-8 (CCK)-8 assay and flow cytometry.

**Results:**

CMT-622 cells maintained stable proliferation beyond 40 passages with a doubling time of 46.23 h and >15% cloning efficiency. Karyotyping revealed hyperdiploidy (80–110 chromosomes; modal = 87). Immunohistochemistry and immunofluorescence confirmed estrogen receptor (+), progesterone receptor (–), and human epidermal growth factor receptor 2 (weak +) expression, consistent with a luminal B phenotype. Co-expression of cytokeratin-18 and vimentin indicated a partial epithelial–mesenchymal transition (EMT) state. In nude mice, CMT-622 exhibited moderate tumorigenicity and pulmonary metastasis. The line showed intermediate osthole sensitivity (half-maximal inhibitory concentration = 50.48 μM) and an apoptosis rate of 21%, between CMT-1211 and CMT-n7, indicating balanced proliferative and drug-responsive behavior.

**Conclusion:**

CMT-622 represents a newly established luminal B CMT cell line with stable growth, EMT plasticity, and moderate drug sensitivity, reflecting clinically relevant tumor aggressiveness. Its molecular and phenotypic consistency across *in vitro* and *in vivo* models underscores its reliability for translational oncology applications. CMT-622 provides a robust preclinical platform for exploring tumorigenesis, metastasis, and therapeutic responses in both veterinary and HBC contexts, bridging comparative and translational cancer research.

## INTRODUCTION

The annual incidence of canine mammary tumors (CMTs) is estimated to be approximately 16.8%, accounting for 50–70% of all neoplasms in intact female dogs and ranking among the most significant neoplastic diseases in canines. Their clinical importance lies in their high frequency, marked histological diversity, aggressive invasive behavior, and frequent metastatic potential [1, 2]. The most common clinical presentation is the occurrence of palpable nodules or masses within the mammary gland [3], often accompanied by secondary signs such as skin ulceration, nipple discharge, and localized pain [4]. As the disease advances, systemic manifestations may appear due to metastatic dissemination [5].

The etiology of CMTs remains incompletely understood [6], though evidence supports a multifactorial pathogenesis influenced by genetic, endocrine, environmental, and immunological factors [7]. Breed predisposition has been well established, with notable variation in susceptibility among different canine breeds [8]. Intact females are significantly more prone to developing mammary tumors than spayed counterparts, and tumor onset typically occurs later in life among unspayed dogs [9]. Estrogen plays a central role by promoting mammary epithelial cell proliferation through ligand-dependent activation of estrogen receptors (ERs) [10], while progesterone may further potentiate tumorigenesis through synergistic interactions with estrogenic pathways under specific physiological conditions [11].

CMTs exhibit remarkable histological and molecular similarities to human breast cancer (HBC) [12]. In particular, the luminal B subtype serves as a high-fidelity comparative model, closely reflecting the molecular signatures, pathological patterns, and clinical aggressiveness of its human counterpart. Despite this, most available CMT cell lines correspond to triple-negative or human epidermal growth factor receptor 2 (HER-2)-overexpressing phenotypes, leaving luminal A and B subtypes underrepresented. Given these parallels, CMT-derived cell lines represent valuable *in vitro* platforms for studying tumor biology, evaluating anticancer agents, and exploring personalized therapeutic strategies [13].

These cell lines bridge the translational gap between conventional human models and clinical reality through their inherent genomic heterogeneity, natural therapy-resistant traits, and conserved tumor microenvironmental features [14]. Since the 1980s, efforts to establish CMT cell lines have intensified. Early foundational work generated CMT-1–CMT-6 using primary culture techniques, which revealed notable differences in morphology, proliferation dynamics, and biomarker expression [15]. For example, the CMT-1 line, derived from a complex-type adenocarcinoma, exhibited myoepithelial characteristics (α-SMA [alpha-smooth muscle actin] and p63 positive), whereas CMT-U27 and CMT-U309 demonstrated high invasiveness and contractility linked to actin bundle abundance and activation of the AMP-activated protein kinase (AMPK) signaling pathway [16]. Recent advancements have yielded new models such as the triple-negative CMT-7364 line [17] and the epithelial–mesenchymal transition (EMT)-characterized FR37-CMT line [18], which collectively enhance the available repertoire of subtype-specific resources. Furthermore, multi-omics analyses have revealed shared dysregulation of miR-200 family non-coding RNAs and glutaminolysis pathways between the CMT line IPC-366 and the HBC line SUM149, reinforcing the translational value of these cross-species models [19].

Despite extensive research on CMTs, the availability of well-characterized *in vitro* models that accurately represent distinct molecular subtypes remains limited. Among these, Luminal B CMTs are particularly underrepresented, even though this subtype is associated with high proliferative activity, partial hormone dependence, and resistance to endocrine therapy, features that mirror human luminal B breast cancer. Existing CMT cell lines predominantly belong to the triple-negative or HER-2-overexpressing categories, such as CMT-7364 and FR37-CMT, which, while valuable, fail to capture the intermediate hormonal and proliferative phenotype characteristic of luminal B tumors. Furthermore, few CMT-derived cell lines have been comprehensively analyzed for their EMT status, clonogenic potential, and *in vivo* metastatic behavior, parameters that are critical for modeling tumor heterogeneity and therapeutic response.

In addition, the lack of standardized biological characterization, including receptor expression, chromosomal stability, and drug sensitivity profiles, limits cross-study comparability and restricts the translational use of these cell lines for preclinical screening. There remains a clear need for establishing new, stable, and biologically defined CMT cell lines that recapitulate both the morphological and molecular complexity of luminal B breast cancer. Such models would enable mechanistic exploration of tumorigenesis, metastasis, and drug resistance across species, thereby strengthening the comparative oncology framework that connects veterinary and human cancer research.

This study aimed to establish and comprehensively characterize a novel CMT cell line, designated CMT-622, derived from a naturally occurring high-grade mammary carcinoma in an unspayed female dog. The primary objectives were to:

1. Isolate, culture, and maintain a stable cell line from spontaneous CMT tissue and confirm its epithelial origin through histopathological and immunohistochemical analyses.
2. Determine molecular subtype classification based on ER, PR, and HER-2 receptor status to identify its alignment with luminal B breast cancer.
3. Assess cellular morphology, growth kinetics, and chromosomal composition to establish the proliferative characteristics and genomic stability of the cell line.
4. Evaluate clonogenic efficiency and tumorigenicity using in vitro colony-forming assays and *in vivo* xenograft models in nude mice to confirm malignancy and metastatic potential.
5. Compare drug sensitivity and apoptosis responses of CMT-622 with previously established CMT-1211 and CMT-n7 cell lines using osthole as a representative antitumor agent.
6. Validate the translational potential of CMT-622 as a reproducible luminal B CMT model suitable for drug testing, molecular pathway studies, and cross-species oncological research.

By fulfilling these objectives, this study contributes a newly established, biologically stable, and phenotypically distinct luminal B CMT model that addresses the current deficit in cell lines representative of this clinically important subtype and provides a foundation for future comparative and translational cancer research.

## MATERIALS AND METHODS

### Ethical approval

All experimental procedures involving animals were conducted in accordance with the ethical standards of the Institutional Animal Care and Use Committee of Anhui Agricultural University, Hefei, China (Approval No.: SYXK [Zhe] 2024-0061; Production License: SCXK [Zhe] 2024-0004). The collection and use of canine tumor tissues were performed with the written informed consent of the owner and under the supervision of licensed veterinarians following institutional and national guidelines.

All *in vivo* experiments involving mice were carried out in a specific pathogen-free (SPF) facility at the Anhui Agricultural University Laboratory Animal Center, adhering to the NIH Guide for the Care and Use of Laboratory Animals (8^th^ edition, 2011). Humane endpoints were predefined, and all efforts were made to minimize pain, distress, and the number of animals used.

This study was conducted and reported in full compliance with the Animal Research: Reporting of *In Vivo* Experiments (ARRIVE) 2.0 guidelines to ensure transparency, reproducibility, and ethical integrity in animal research.

### Study period and location

The study was conducted from September 2023 to December 2024 at the Department of Traditional Chinese Veterinary Medicine, College of Animal Science, Anhui Agricultural University.

### Reagents

Dulbecco’s Modified Eagle Medium (DMEM), Cell Counting Kit-8 (CCK8), and Trypsin (0.25%) were purchased from Biosharp Life Sciences Co., Ltd. (Hefei, China). Osthole (HPLC ≥98%, CAS#: 484-12-8) was procured from Shanghai Yuanye Bio-Technology Co. Ltd. (Shanghai, China). Penicillin-streptomycin (P/S) (BC-CE-007) was purchased from Bio-Channel (Shenzhen, China). Collagenase (C8140) and Crystal Violet Staining Solution (G1064) were purchased from Solarbio (Beijing, China). CK18 (10830-1-AP), progesterone receptor (PR) (25871-1-AP), ER (21244-1-AP), and p53 (10442-1-AP) were procured from Wuhan SunnyBio Technology Co., Ltd. (Wuhan, China). Ki-67 (ab16667), HER-2 (ab134182), and Vimentin (ab92547) were procured from Abcam (Shanghai, China). Fetal Bovine Serum (100% FBS, FBS500-H) was procured from Hycezmbio (Wuhan, China). Phosphate-buffered saline (PBS) and Citrate Antigen Retrieval Solution were procured from Zhongshan Goldenbridge Biotechnology (Beijing, China). More material information and antibody dilution concentrations are presented in Table 1.

**Table 1:** Information on the antibodies used in this study.

Antibodies	Product code	Dilution	Species	Manufacturer
Ki-67	ab16667	1:200 (IF, IHC)	Rabbit	Abcam
HER-2	ab134182	1:250 (IF, IHC)	Rabbit	Abcam
Vimentin	ab92547	1:500 (IF, IHC)	Rabbit	Abcam
CK18	10830-1-AP	1:800 (IF, IHC)	Rabbit	ProteinTech Group
PR	25871-1-AP	1:500 (IF, IHC)	Rabbit	ProteinTech Group
ER	21244-1-AP	1:500 (IF, IHC)	Rabbit	ProteinTech Group
p53	10442-1-AP	1:800 (IF, IHC)	Rabbit	ProteinTech Group

Ki-67 = Nuclear-associated antigen (NAA), CK18 = Cytokeratin 8, PR = Progesterone receptor, ER = Estrogen receptor, HEG-2 = Human epidermal growth factor receptor 2, p53 = Tumor protein p53, IHC = Immunohistochemistry, IF = Immunofluorescence.

### Cell counting

The hemocytometer and coverslip were cleaned with 95% ethanol and gently wiped dry. A single-cell suspension was prepared from the digested cells and gently mixed using a Pasteur pipette. A small amount of the suspension (1–2 drops) was added to one side of the coverslip on the hemocytometer. The hemocytometer was placed under a microscope for counting. Using a 10× objective lens, the number of cells in the four large corner squares of the hemocytometer was counted. When cells touched the middle line, only those on the left and upper lines were counted, while those on the right and lower lines were ignored. The obtained results were substituted into the formula to calculate the cell density:

Cells/mL of original suspension = (Sum of cell counts in 4 large squares/4) × 10^4^ × Dilution Factor

### Tumor specimens

CMT specimens were collected from multiple veterinary hospitals in Hefei (2023–2024). The tumor sample was obtained from an 11-year-old, female, unspayed Teddy dog following standard operating procedures and with informed owner consent. Histopathological evaluation classified the tumor as Grade III (highly malignant) and staged it as T3N1M0. Tumor sections with at least one flat cut surface were fixed in 4% paraformaldehyde for histological analysis. The remaining tumor tissues were transferred to a laminar flow cabinet for primary culture. Connective and necrotic tissues were excised, and 1 cm³ tissue blocks were harvested from tumor–normal tissue junctions for cell culture.

### Establishment, maintenance, and purification of cell lines

The CMT-622 cell line was established from primary tumor tissues. Briefly, the minced tissues were digested with collagenase I (1 mg/mL) for 2 h at 37°C in a 5% CO_2_ incubator. The digested slurry was filtered and centrifuged, and the isolated cells were initially cultured in DMEM supplemented with 20% FBS and 1% P/S to facilitate primary cell attachment and growth. Near-confluent cells were passaged using 0.25% trypsin-ethylenediaminetetraacetic acid for routine subculturing, with enzyme activity neutralized by the standard culture medium (DMEM containing 10% FBS and 1% P/S). The cell line has been successfully maintained for more than 40 generations.

Cells were suspended in a cryoprotective medium consisting of 90% FBS and 10% dimethyl sulfoxide, slowly frozen at –80°C, and subsequently transferred to liquid nitrogen for long-term storage. Thawing was performed rapidly in a 37°C water bath, followed by dilution in pre-warmed complete medium and subsequent culturing.

Following digestion, a single-cell suspension was prepared and centrifuged at 1,000 × g for 5 min. The supernatant was discarded, and the cell pellet was resuspended in serum-free medium. The suspension was incubated in a cell culture incubator for 15–25 min. Non-adherent cells were gently aspirated in their entirety and transferred to a new T25 flask for continued culture.

### Growth assay and population doubling time

Cells in the logarithmic phase at passage 27 were trypsinized, counted, and uniformly seeded into 21 wells of a 24-well plate, with 3 wells reserved as medium-only controls. Starting from seeding (0 h), the medium was aspirated from 3 wells every 24 h. Cells from these wells were trypsinized and counted in triplicate to determine the mean density (5 × 10³ cells/well). The non-sampled wells received fresh medium every 2–3 days. Sampling continued for 7–10 days until the population declined. Growth curves (time versus cell number) were plotted, and the population doubling time was calculated using nonlinear regression in GraphPad Prism 5 (GraphPad, San Diego, CA, USA). The experiments were performed in triplicate.

### Single-cell cloning

Log-phase cells at passage 27 were trypsinized, counted, and seeded in 6-well plates at gradient densities of 50, 100, and 200 cells/well. Plates were gently swirled crosswise to ensure uniform distribution and incubated statically at 37°C for 2–3 weeks. When macroscopic colonies appeared, the cultures were terminated. After aspirating the medium, the cells were washed twice with PBS, fixed with 2 mL methanol (15 min), and stained with 2 mL crystal violet (3 min). Plates were rinsed under running water, air-dried, and colonies (>50 cells) were manually counted microscopically. All experiments were performed in triplicate. The cell cloning efficiency was calculated using the following formula:

Clone formation rate = (Number of clones / number of inoculated cells) × 100%

### Cytogenetic analysis

At passage 27, cells at 50%–70% confluency were treated with colchicine, trypsinized, and pelleted. The pellet underwent hypotonic KCl treatment, followed by repeated fixation in acetic acid: methanol. Cell suspensions were dropped from a height onto chilled slides, air-dried, and stained with Giemsa (Solarbio, China) for 15 min. Chromosome numbers were observed and counted in 40 metaphase cells under a light microscope (N-300M, NOVEL, Ningbo, China). The assessment focused on numerical abnormalities (aneuploidy), specifically identifying chromosomal gains or losses.

### Histological and immunohistochemical analyses

Tissue sections from specimens were fixed in 10% neutral-buffered formalin for 24 h, dewaxed in xylene, and rehydrated using a graded ethanol series. Antigen retrieval was performed using a high-pressure heating method in pH 9.0 EDTA buffer (2 L) for 2 min. Endogenous peroxidase activity was blocked with 3% H_2_O_2_ for 10 min at room temperature. After washing, the sections were incubated with primary antibodies (Ki-67, HER-2, Vimentin, CK18, PR, ER, and p53) for 60 min at 37°C, followed by incubation with an HRP-conjugated secondary antibody for 30 min at 37°C. Signal detection was performed using a DAB Kit (Kit-5230, Fuzhou Maixin, Fujian, China), and the reaction was microscopically monitored. Sections were counterstained with hematoxylin, dehydrated, cleared in xylene, and mounted with neutral balsam. Negative controls (incubated with PBS instead of the primary antibody) and positive controls (known positive tissue sections) were included.

### Immunofluorescence staining

At passage 27, cell climbing slides were prepared from a suspension of 10^5^ cells/mL for immunofluorescence. After fixation with 4% paraformaldehyde and permeabilization with 0.3% Triton X-100, heat-induced antigen retrieval was performed using preheated EDTA (pH 9.0) or citrate (pH 6.0) buffer. After blocking with goat serum, the slides were incubated with primary antibodies (Ki-67, HER-2, Vimentin, CK18, PR, ER, p53) at 4°C overnight or at 37°C for 1 h, followed by incubation with fluorophore-conjugated secondary antibodies for 30 min at room temperature. Signal amplification was achieved using aTSA fluorescence kit (RK50028P, Wuhan Abbkine Biotechnology Co., Ltd. [Abclonal], Wuhan, China). Nuclei were counterstained with DAPI, and slides were mounted with anti-fade mounting medium. Images were captured using a TG FAXS Plus S fluorescence microscope (TissueGnostics, Austria) and analyzed with Tissue FAXS Viewer software version 8.0.0.158 (TissueGnostics, Austria). Appropriate positive and negative controls were included in each experiment.

### Tumorigenicity in nude mice

Female SPF BALB/c-nu mice (5 weeks old) were purchased from Hangzhou Ziyuan Experimental Animal Technology Co., Ltd, China. Mice (n = 4 per group, including one negative control per group) received a subcutaneous injection in the dorsal flank with either 1.0 × 10^6^ CMT cells (passage 27) in 100 μL PBS or PBS alone. Physiological status and tumor growth were monitored weekly for 30 days. Once tumors were palpable, their length (a) and width (b) were measured using a vernier caliper, and tumor volume was calculated as:

V = a × b^2^ × 0.5 (mm^3^)

Humane endpoints were defined as tumor volume exceeding 1500 mm^3^ or body weight loss >15%. At day 30, mice were euthanized by CO_2_ asphyxiation. Lungs and major organs were collected and examined macroscopically for metastasis. All tissues were fixed in 4% paraformaldehyde, embedded in paraffin, and sectioned for histological and immunohistochemical analysis.

### CCK8 cell viability assay

CMT-622, CMT-1211, and CMT-N7 cells were plated in 96-well plates (10^4^ cells/well; CMT-622 at passage 27) and incubated for 24 h. The medium was replaced with 200 μL of fresh medium containing osthole (0, 10, 25, 50, 75, 100 μM). Blank wells (medium only), control wells (cells with medium), and experimental wells (cells with osthole) were prepared. After 24 h incubation, CCK-8 solution was added under light protection and incubated for 4 h. Absorbance was measured at 450 nm, and cell survival rate and half-maximal inhibitory concentration (IC_50_) values were calculated to assess osthole’s inhibitory effect on cell proliferation:

Cell survival rate (%) = ([As − Ab] / [Ac − Ab]) × 100%

Where, As = Experimental Well: Culture medium containing cells, CCK-8 reagent, and the drug. Ac = Control Well: Culture medium containing cells and CCK-8 reagent, but no drug. Ab = Blank Well: Culture medium containing neither cells nor the drug, with CCK-8 reagent.

### Detection of apoptotic response

Flow cytometry was conducted using a CytoFLEX instrument (Beckman, Waltham, MA, USA). The excitation laser was set at 488 nm, and fluorescence was detected using 525/40 b and pass (BP) and 585/42 BP for fluorescein isothiocyanate (FITC) and phycoerythrin (PE) channels, respectively. Data were analyzed using FlowJo V10 software. The gating strategy was defined based on negative controls, and gates were drawn according to population distribution.

CMT-622 (passage 27), CMT-1211, and CMT-N7 cells were treated with 50 μM osthole at 50%–70% confluence for 24 h. Harvested cells were resuspended in binding buffer (5 × 10^5^ cells/mL), stained with Annexin V-FITC (V-fluorescein isothiocyanate) and PI (propidium iodide), and incubated in the dark for 5 min. Apoptosis was evaluated according to Annexin V/PI fluorescence intensities.

### Mycoplasma test

To detect Mycoplasma contamination, both cell culture supernatant and cell pellets were collected at passage 27. After 48–72 h of culture, 10 μL of supernatant was used as a polymerase chain reaction (PCR) template. Genomic DNA was extracted from the cell pellet using a blood/cell/tissue DNA extraction kit. The PCR reaction included negative (MycoFree H_2_O) and positive controls. PCR products were analyzed by electrophoresis on a 1%–1.5% agarose gel at 160 V for 15 min. The gel was imaged, and samples were interpreted by comparison with controls. A positive band at approximately 250 base pair indicated mycoplasma contamination.

### Statistical analysis

Quantitative data are presented as mean ± SEM from at least three independent experiments. Statistical analyses were performed using GraphPad Prism 5 (GraphPad). Differences between the two groups were analyzed using a two-tailed unpaired Student’s t-test. For multiple group comparisons, one-way ANOVA followed by Dunnett’s *post hoc* test was applied. Statistical significance was considered at *p* < 0.05.

## RESULTS

### Identification of breast tumors and establishment of the CMT-622 primary cell line

**Figure 1:**
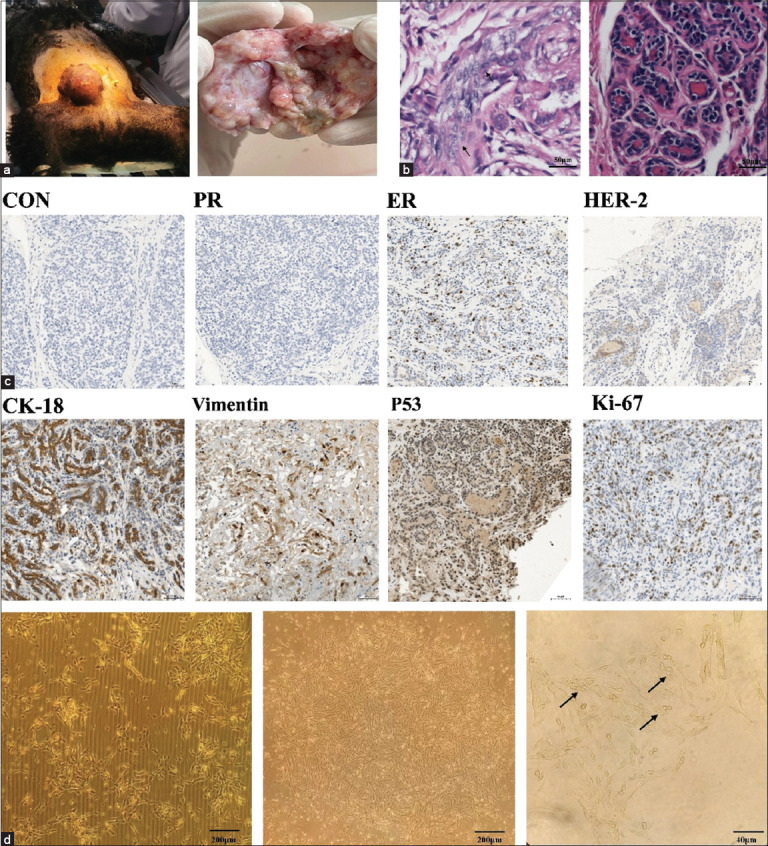
(a) Morphology of primary canine mammary tumors. (b) Moderate pleomorphism of tumor cells, and nuclear division is more easily seen (indicated by black arrows); normal structure of mammary lobule. (c) Negative control. The primary tumor tissue is negative for progesterone receptor, weakly positive for human epidermal growth factor receptor 2, positive for estrogen receptor, positive for CK18 and Vimentin on the cell membrane, and positive for p53 and Ki-67 in the cell nucleus. (d) On the left, after 48 h of primary culture, the cells swam out and grew attached to the wall; on the middle, the cells covered the bottom of the flask and grew overlappingly; on the right, the cells were spindle-shaped and had epithelial morphology of malignant tumor cells; the nucleoli were enlarged and increased in number, the nuclear-cytoplasmic ratio was abnormally increased, and highly malignant multinucleated cells were observed (as shown by the arrow on the right). Positive staining is observed as dark brown. Ki-67 = Nuclear-associated antigen (NAA), CK18 = Cytokeratin 8, p53 = Tumor protein p53.

From 30 primary CMT cultures, the CMT-622 cell line was successfully established and maintained for over 40 passages. The cell line originated from an intact, 11-year-old female Teddy dog. Gross examination revealed a firm, irregularly shaped mammary mass with nodular protrusions (Figure 1a). Histopathological examination showed disorganized tumor cells with marked nuclear enlargement, hyperchromasia, and frequent mitotic figures, confirming the diagnosis of mammary carcinoma (Figure 1b).

Immunohistochemical analysis of the primary tumor demonstrated ER positivity, PR negativity, and weak HER-2 membrane staining (Figure 1c). Cytokeratin-18 (CK-18) expression confirmed mammary epithelial origin, while vimentin positivity indicated EMT potential. High nuclear Ki-67 reactivity reflected strong proliferative activity, and nuclear accumulation of p53 suggested tumor-suppressor dysfunction. Based on these molecular features, the tumor was classified as luminal B subtype.

Following enzymatic digestion of the tumor tissue, adherent cells were purified using differential adhesion (Figure 1d). The established CMT-622 cells displayed neoplastic characteristics, including loss of contact inhibition, multilayered growth, and nuclear atypia characterized by enlarged, irregular nuclei with prominent nucleoli. Subpopulations with spindle-shaped morphology and elongated eccentric nuclei were also observed, indicating cellular heterogeneity. The cell line exhibited stable proliferation beyond 40 passages.

### Comprehensive phenotypic profiling of CMT-622: Mycoplasma testing, proliferation, clonogenicity, immunophenotype, and genomic instability

**Figure 2:**
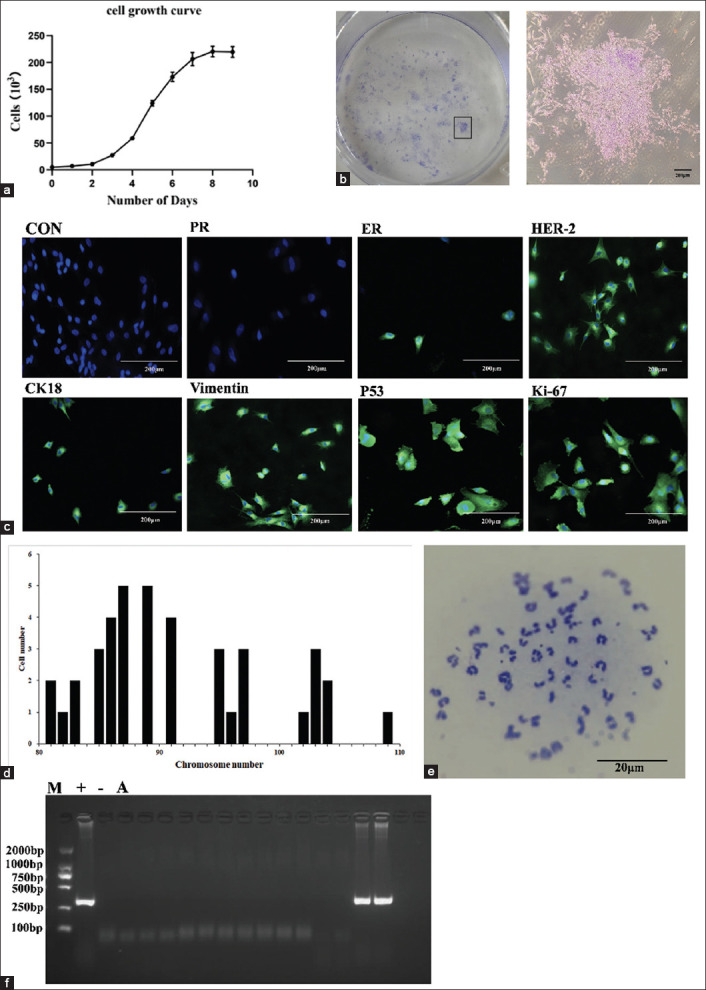
(a) Growth curve of the canine mammary tumors (CMT)-622 cell line; (b) colony-forming ability of CMT-622 cells (n = 3); (c) negative for progesterone receptor, positive for estrogen receptor and human epidermal growth factor receptor 2, and positive for CK18 and vimentin. The positive cells are located on the cell membrane, and those positive for p53 and Ki-67 are in the nucleus. (d) The range of chromosome numbers in the cell lines. (e) Abnormal chromosome numbers (n = 3). (f) Mycoplasma detection. The CMT-622 cell line exhibited no Mycoplasma contamination. M, maker; +, positive control; –, negative control; A, CMT-622 cells (n = 3). Ki-67 = Nuclear-associated antigen (NAA), CK18 = Cytokeratin 8, p53 = Tumor protein p53.

The CMT-622 cell line exhibited a characteristic S-shaped growth curve (Figure 2a), comprising lag, logarithmic, and plateau phases. The population doubling time was calculated as 46.32 h, and cryopreserved cells retained their proliferative potential upon recovery. Clonogenic assays demonstrated a high colony-forming efficiency (>15%), with the development of large, compact colonies within 2 weeks (Figure 2b).

Immunofluorescence staining (Figure 2c) confirmed the expression profile consistent with luminal B phenotype: ER and HER-2 positivity, PR negativity, membranous CK-18 and vimentin expression, and nuclear localization of Ki-67 and p53. This immunophenotype closely mirrored that of the original primary tumor, confirming cellular authenticity.

Karyotype analysis of 40 metaphase spreads revealed chromosomal hyperdiploidy (80–110 chromosomes/cell) with a modal count of 87 chromosomes (Figures 2d and e), indicating extensive aneuploidy and genomic instability. Mycoplasma testing by PCR verified that the CMT-622 cells were free from Mycoplasma contamination (Figure 2f).

### Histopathological characterization of xenograft tumors and pulmonary metastasis in nude mice

**Figure 3:**
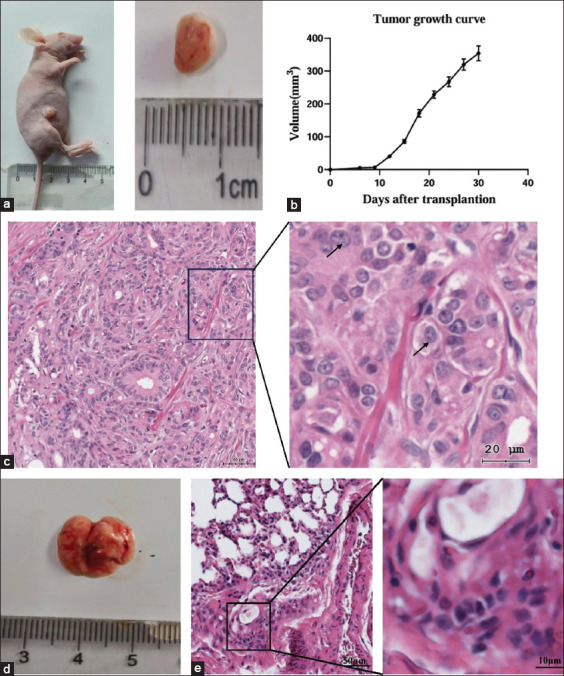
(a) Subcutaneous injection of 5 × 10^6^ cells into nude mice resulted in rapid tumor growth and the formation of subcutaneous tumors. (b) Growth curve of xenograft tumors over time. (c) Histopathological observation of xenograft tumors. (d) Visual visualization of lung tissue with a prominent hard parenchymal tissue. (e) The microscopic structure of the lung tissue is disordered, the alveolar structure is partially destroyed and disappears, and the cells aggregate into nests. Local magnification reveals marked nuclear pleomorphism, hyperchromatic (darker staining), and anisocytosis (variation in nuclear size).

To evaluate tumorigenicity, CMT-622 cell suspensions were subcutaneously inoculated into two groups of 5-week-old female nude mice (n = 3/group). Palpable nodules developed at the injection sites by day 7 in 2 of 6 mice, indicating slow initial tumor progression. Rapid tumor growth began around day 15, and by day 30, the mean tumor volume reached 356 mm³. Mice were euthanized by carbon dioxide asphyxiation on day 30 in accordance with Institutional Animal Care and Use Committee protocols. Visible tumor vasculature was documented (Figure 3a), and 1 of 6 mice showed pulmonary metastasis. Tumor growth kinetics were plotted based on caliper measurements (Figure 3b).

Histopathological examination of xenografted tumors revealed disorganized architecture with spindle-shaped cells arranged in whorled patterns, accompanied by stromal fibrosis and hyperplasia. Neoplastic cells showed pronounced nuclear atypia, including pleomorphism, coarse chromatin clumping, and prominent nucleoli. Focal findings included nuclear enlargement, hyperchromasia, and atypical mitoses (Figure 3c). These features closely resembled the primary CMT, confirming species fidelity.

Gross examination of the lungs revealed distinct white metastatic nodules (Figure 3d). Microscopic sections demonstrated cellular aggregates forming irregular nests that disrupted the alveolar structure (Figure 3e). Pronounced nuclear pleomorphism and hyperchromasia were observed, consistent with metastatic involvement characterized by alveolar obliteration and septal thickening.

### Immunohistochemical characterization of xenografts and comparative clonogenic analysis

**Figure 4:**
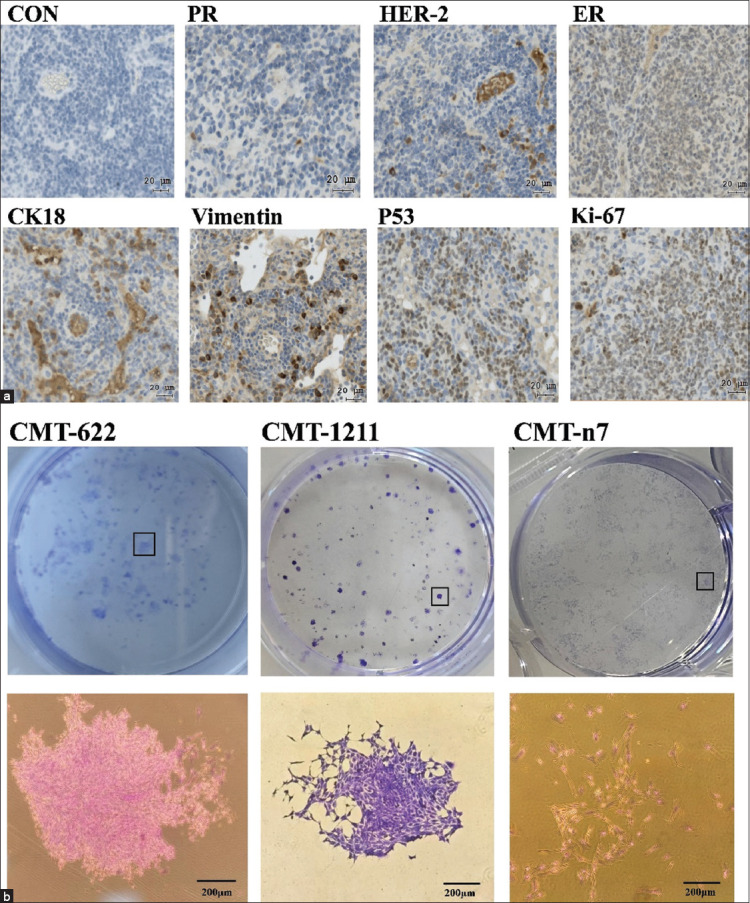
(a) The tissue of the xenograft tumor is negative for progesterone receptor, weakly positive for human epidermal growth factor receptor 2, positive for estrogen receptor, positive for CK18 and Vimentin on the cell membrane, and positive for p53 and Ki-67 in the cell nucleus. (b) Clonal colony formation and morphology of canine mammary tumors (CMT)-622, CMT-1211, and CMT-n7 (n = 3). Ki-67 = Nuclear-associated antigen (NAA), CK18 = Cytokeratin 8, p53 = Tumor protein p53.

Immunohistochemical staining of xenograft tumor sections confirmed that the transplanted CMT-622 tumors retained the same molecular profile as the original lesion. Xenograft tissues showed PR negativity, weak HER-2 positivity, and ER positivity. CK-18 and vimentin were strongly expressed along cell membranes, while Ki-67 and p53 were localized to the nuclei (Figure 4a). This immunophenotype was consistent with both the primary tumor and the *in vitro* cultured CMT-622 cells, indicating stable molecular characteristics *in vivo*.

A comparative clonogenicity assay was performed using CMT-622, CMT-1211, and CMT-n7 cell lines to evaluate colony-forming capacity (Figure 4b). The results demonstrated distinct differences among the three lines. CMT-622 exhibited strong and stable colony-forming ability, characterized by numerous compact colonies with uniform cell morphology. CMT-1211 showed moderate clonogenicity, forming looser colonies with larger intercellular spaces. CMT-n7 had the lowest clonogenic potential, forming few irregular colonies with heterogeneous morphology and unstable structure. Overall, CMT-622 demonstrated superior proliferation and colony formation potential compared to CMT-1211 and CMT-n7.

### Comparative drug sensitivity and apoptotic response among CMT cell lines

**Figure 5:**
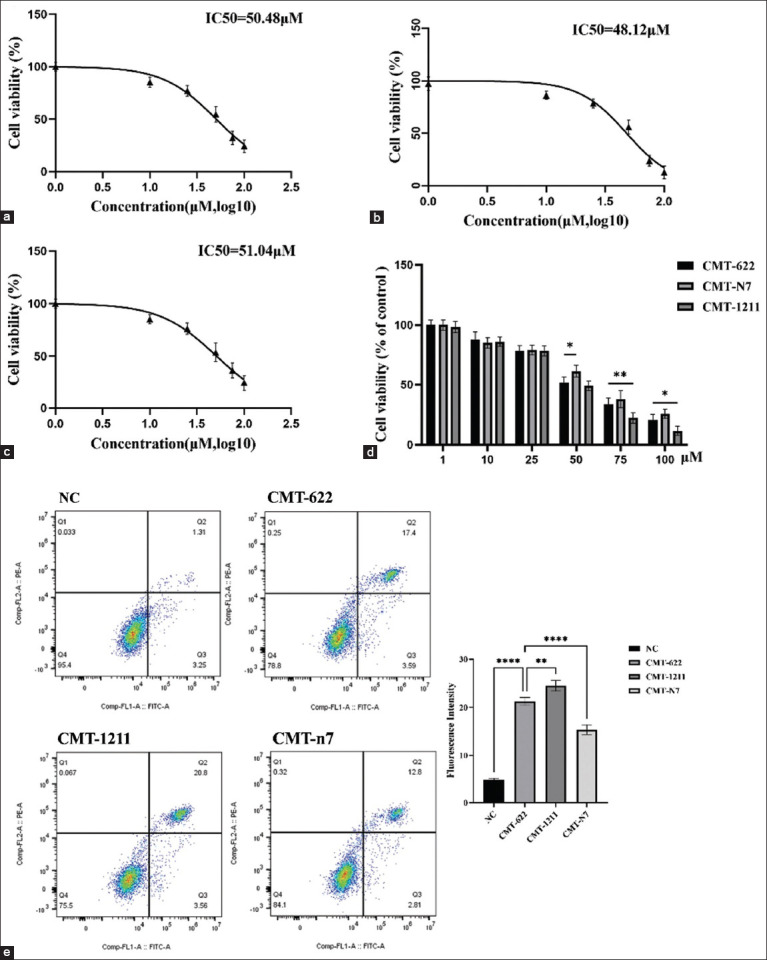
(a) Half-maximal inhibitory concentration (IC_50_) curve of canine mammary tumors (CMT)-622 cell line; (b) IC_50_ curve of CMT-1211 cell line; (c) IC_50_ curve of CMT-n7 cell line. (d) Comparison of the survival rates of CMT-622, CMT-n7, and CMT-1211 cell lines at different osthole concentrations. (e) Flow cytometry analysis of cell lines (NC group, CMT-622, CMT-1211, and CMT-n7) treated with 50 μM osthole revealed distinct apoptotic responses. CMT-1211 exhibited the highest total apoptosis rate (24.4%), followed by CMT-622 (21%), while CMT-n7 showed the lowest susceptibility (15.6%). Data are presented as mean ± standard deviation (n = 3) in the corresponding figures. The quadrant definitions were as follows: Q4, viable cells/normal cells; Q3, early apoptotic cells; Q2, late apoptotic cells; and Q1, necrotic cells (*p < 0.05, **p < 0.01 and ****p < 0.0001).

Drug sensitivity to osthole was compared among CMT-622, CMT-1211, and CMT-n7 cell lines. The calculated IC_50_ values were 50.48 μM (CMT-622; Figure 5a), 48.12 μM (CMT-1211; Figure 5b), and 51.04 μM (CMT-n7; Figure 5c), indicating intermediate sensitivity of CMT-622 relative to the other two lines.

Cell viability curves (Figure 5d) demonstrated comparable survival rates at low osthole concentrations but significant divergence at higher doses. Above 50 μM, CMT-622 exhibited lower survival than CMT-n7 but higher survival than CMT-1211 at 75 μM and 100 μM (p < 0.01).

At the 50 μM IC_50_ concentration, apoptosis was analyzed by flow cytometry (Figure 5e). The apoptosis rates were 24.4% (CMT-1211), 21.0% (CMT-622), and 15.6% (CMT-n7). Statistical comparison revealed significantly higher apoptosis in CMT-1211 versus CMT-622 (p < 0.01) and an even greater difference between CMT-622 versus CMT-n7 (p < 0.001).

These results were consistent with the IC_50_ data, indicating that CMT-1211 was the most sensitive, CMT-n7 was the least sensitive, and CMT-622 demonstrated intermediate drug sensitivity and apoptotic response.

## DISCUSSION

### Role of tumor cell lines in cancer research

Tumor cell lines serve as essential tools in oncology research. They facilitate the investigation of molecular mechanisms underlying tumorigenesis and progression, enable anticancer drug screening and biomarker discovery, and support the validation of new experimental techniques [20]. In this study, a single stable CMT cell line, CMT-622, was successfully established from 30 primary cultures derived from female dogs with spontaneous mammary tumors. The cell line was continuously propagated through serial passages and maintained stable growth characteristics.

Tumor cell purification was achieved using differential adhesion, leveraging the slower attachment kinetics of tumor cells compared to fibroblasts. Sequential adhesion cycles in serum-free medium effectively isolated both populations, consistent with the method described by Jin *et al*. [21]. This technique ensured the enrichment of neoplastic epithelial cells while minimizing fibroblast contamination.

### Growth characteristics and cytogenetic features of CMT-622

The growth kinetics of the CMT-622 line were evaluated across three distinct phases: initiation (day 0), logarithmic growth, and plateau. Cells entered exponential proliferation by day 2 post-inoculation, sustained logarithmic growth for approximately 5 days, and reached a plateau due to contact inhibition and nutrient depletion. These kinetics are consistent with previous observations for the CMT-7364 cell line [22].

Chromosomal analysis revealed a range of 138–174 chromosomes per cell, representing significant aneuploidy relative to the normal canine karyotype (78 chromosomes) and indicating marked karyotypic heterogeneity. Such genomic instability is a hallmark of canine tumorigenic cell lines, reflecting their underlying genetic volatility and tumorigenic potential.

### Immunophenotypic characterization and molecular subtype identification

The CMT-622 cell line retained the original immunophenotype of the primary tumor, exhibiting similar protein expression profiles across the primary tissue, established cell line, and mouse xenograft. Specifically, the cells were ER positive, PR negative, and weakly positive for HER-2, while CK18, vimentin, p53, and Ki-67 were strongly expressed.

These biomarker profiles align with the luminal B subtype classification of CMT, characterized by partial hormone dependence, HER-2 positivity, and high proliferative activity [23]. This receptor expression pattern is clinically significant for early diagnosis, therapeutic decision-making, and prognostic assessment in CMT. Immunohistochemical and immunofluorescent analyses confirmed ER and HER-2 positivity, PR negativity, and strong Ki-67 expression, supporting the luminal B classification.

By establishing a well-defined and genetically stable luminal B CMT model, this study addresses the limitations of existing cell lines that often fail to reproduce luminal tumor heterogeneity or endocrine resistance, distinguishing CMT-622 from previously reported models such as CMT-1026 and CMT-7364 [22, 24].

### Tumorigenicity and metastatic potential *in vivo*

The *in vivo* tumorigenicity and metastatic capability of CMT-622 were confirmed using a nude mouse xenograft model. Tumor nodules were palpable within 7 days post-subcutaneous inoculation, with rapid volume increase after 2 weeks. Histopathological examination (hematoxylin and eosin staining) revealed that the xenografts reproduced the structural and cytological characteristics of the primary tumor, including anisocytosis, mitotic activity, and angiogenesis. Furthermore, tumor cell infiltration into lung tissue was observed in several mice, corroborating previous reports of metastatic behavior in CMT cell lines [25, 26]. These findings validate the malignant phenotype and metastatic potential of CMT-622.

### Evidence of partial EMT

Expression of CK18 (epithelial marker) and vimentin (mesenchymal marker) is typically mutually exclusive; however, during tumor progression, cancer cells may simultaneously express both markers, signifying partial EMT [27]. In the xenograft tumors, co-expression of CK18 and vimentin confirmed the presence of this hybrid phenotype.

The partial EMT state in CMT-622 suggests that the *in vivo* microenvironment, through inflammatory and hypoxic cues, supports cellular plasticity, enabling survival, invasion, and drug resistance. This hybrid phenotype recapitulates the morphological and functional features of the primary tumor and provides a clinically relevant model for studying metastasis and therapy resistance in both canine and human luminal B breast cancers.

### Role of Ki-67 and p53 in proliferation and prognosis

Ki-67 is a nuclear proliferation marker widely used for molecular classification and prognostic stratification in breast cancer [28]. Its expression is influenced by factors such as tumor size, inflammation, invasion, and lymph node involvement [29]. The high Ki-67 expression observed in CMT-622 correlates with its rapid *in vitro* proliferation, supporting its use as a robust continuous cell line for long-term studies and high-throughput drug screening.

The p53 tumor suppressor protein is closely associated with tumor grade, metastasis, and clinical outcome [30]. Brunetti *et al*. [31] reported that p53 positivity correlates with poor prognosis in dogs. Although p53 is an established prognostic marker in HBC, its relevance in CMTs remains underexplored [32]. The high p53 expression detected in CMT-622 suggests dysregulated tumor-suppressive signaling, providing a valuable platform to investigate p53-mediated pathways in CMT pathogenesis and to evaluate its potential as a therapeutic target.

### Clonogenic potential and functional EMT plasticity

In clonogenic assays, approximately 30% of single-cell inoculations of CMT-622 formed colonies within 10 days, slightly exceeding that of the IMC-118 canine inflammatory mammary carcinoma line [33]. This high clonogenic efficiency reflects the co-expression of epithelial (CK18) and mesenchymal (vimentin) markers, indicative of a hybrid EMT state.

This hybrid phenotype enhances cellular plasticity, facilitating adaptation to microenvironmental stress and promoting survival and metastasis [34]. Compared with CMT-1211 and CMT-n7, CMT-622 exhibited greater colony compactness and formation efficiency, indicating superior self-renewal and survival capabilities.

### Mechanisms underlying intermediate drug sensitivity

CMT-622 demonstrated moderate drug sensitivity and intermediate apoptosis rates, positioned between the highly sensitive CMT-1211 and resistant CMT-n7 lines. This intermediate profile may result from a balance between intrinsic drug resistance and apoptosis suppression. Possible contributing mechanisms include partial expression of drug efflux transporters (e.g., P-glycoprotein), B-cell lymphoma (Bcl-2) overexpression, impaired p53-dependent apoptosis, and the existence of a stem-like subpopulation.

These factors collectively shape the unique sensitivity phenotype of CMT-622, providing a translationally relevant model for drug screening. The clonogenic capacity of tumor cells is regulated by interlinked signaling pathways, such as PI3K/AKT, Wnt/β-catenin, and Notch, which cooperate to sustain proliferation and self-renewal [35–37]. Activation of c-Myc and suppression of p53 further enhance clonogenic potential [38]. Moreover, transforming growth factor beta signaling modulates these effects in a stage-dependent manner.

The superior clonogenicity and compact colony morphology of CMT-622 may therefore reflect coordinated activation of these oncogenic pathways. In contrast, the high drug sensitivity of CMT-1211 may relate to more efficient drug uptake and activation of AKT/mTOR signaling [39], while the reduced sensitivity of CMT-622 could involve stronger cell cycle regulation, attenuated apoptosis, and enhanced drug efflux. Nonetheless, its moderate response remains advantageous for evaluating drug efficacy across variable resistance contexts. In addition, regulatory mechanisms such as autophagy may further influence drug response dynamics [40].

## CONCLUSION

In this study, a novel CMT cell line, designated CMT-622, was successfully established and comprehensively characterized from a spontaneous high-grade mammary carcinoma in an unspayed 11-year-old female dog. The cell line exhibited stable proliferation beyond 40 passages, with a doubling time of approximately 46.32 h, and maintained a luminal B-like immunophenotype characterized by ER positivity, PR negativity, and weak HER-2 expression. Cytogenetic analysis revealed hyperdiploidy (80–110 chromosomes; modal number = 87) and marked aneuploidy, indicating genomic instability typical of malignant cells. The immunohistochemical and immunofluorescent profiles were consistent among the primary tumor, *in vitro* culture, and xenograft, confirming cellular authenticity and stability. In nude mice, CMT-622 demonstrated tumorigenicity and metastatic potential, with palpable tumors forming within 7 days post-inoculation and pulmonary metastasis observed in one animal.

The co-expression of CK18 and vimentin indicated a partial EMT phenotype, reflecting tumor plasticity and adaptability to the *in vivo* microenvironment. The cell line also showed moderate sensitivity to osthole (IC_50_ = 50.48 μM) and an intermediate apoptotic response (21%), positioning it between the more sensitive CMT-1211 and more resistant CMT-n7 cell lines. These findings confirm that CMT-622 represents a genetically unstable, phenotypically heterogeneous, and clinically relevant luminal B CMT model that captures key features of tumor aggressiveness, heterogeneity, and partial EMT.

The establishment of CMT-622 provides a practical and reproducible platform for exploring the molecular mechanisms of tumorigenesis, metastasis, and endocrine resistance in luminal B mammary cancers. Its intermediate drug sensitivity and EMT-associated plasticity make it particularly suitable for drug screening, mechanistic studies, and biomarker validation in both veterinary and human oncology. The principal strength of this study lies in the comprehensive characterization of a stable luminal B CMT line verified through histopathology, cytogenetics, immunophenotyping, and *in vivo* validation, thereby bridging a major gap in existing CMT model systems.

Nevertheless, this study is limited by the use of a single patient-derived tumor and the absence of *in vivo* therapeutic validation. Moreover, the *in vitro* culture system cannot fully replicate the complex tumor–stroma and immune interactions that govern EMT and drug responses. Future research should therefore expand the model repository through multi-source derivations, integrate 3D organoid or co-culture systems, and employ multi-omics approaches to elucidate the key signaling pathways, such as PI3K/AKT, Wnt/β-catenin, and Notch, that regulate proliferation, EMT, and chemoresistance.

In conclusion, CMT-622 represents a novel, biologically stable, and translationally relevant luminal B CMT cell line that bridges comparative and human oncology. Its combined features of genomic instability, EMT plasticity, and intermediate drug responsiveness make it a valuable tool for mechanistic investigations, anticancer drug development, and One-Health-oriented cancer research aimed at improving therapeutic strategies across species.

## AUTHORS’ CONTRIBUTIONS

TZ, SM, and QP: Supervised the study, administration, and drafted and edited the manuscript. QP, JS, and ZL: Investigation, formal analysis, and data curation. SM and QZ: Methodology, formal analysis, and investigation. TZ: Supervision, project administration, and conceptualization. All authors have read and approved the final manuscript.
